# Upper Limb Function but Not Proprioception is Impaired in Essential Tremor: A Between-Groups Study and Causal Mediation Analysis

**DOI:** 10.5334/tohm.731

**Published:** 2023-01-03

**Authors:** Annie A. Butler, Joanna Diong, Kajsa Lidman, Johanna Adler, Daniel L. Wardman, Simon C. Gandevia, Martin E. Héroux

**Affiliations:** 1 Neuroscience Research Australia, Randwick, 2031, NSW, Australia; 2 School of Medical Sciences, University of New South Wales, Randwick, 2031, NSW, Australia; 3 School of Medical Sciences, Faculty of Medicine and Health, The University of Sydney, 2006, Sydney, NSW, Australia; 4 Faculty of Medicine and Health Sciences, Linköping University, SE-581 83 Linköping, Sweden

**Keywords:** essential tremor, proprioception, function, causal mediation, upper limb

## Abstract

**Background:**

Essential tremor (ET) is characterized by abnormal oscillatory muscle activity and cerebellar involvement, factors that can lead to proprioceptive deficits, especially in active tasks. The present study aimed to quantify the severity of proprioceptive deficits in people with ET and estimate how these contribute to functional impairments.

**Methods:**

Upper limb sensory, proprioceptive and motor function was assessed inindividuals with ET (n = 20) and healthy individuals (n = 22). To measure proprioceptive ability, participants discriminated the width of grasped objects and the weight of objects liftedwith the wrist extensors. Causal mediation analysis was used to estimate the extentthat impairments in upper limb function in ET was mediated by proprioceptive ability.

**Results:**

Participants with ET had impaired upper limb function in all outcomes, and had greater postural and kinetic tremor. There were no differences between groups in proprioceptive discrimination of width (between-group mean difference [95% CI]: 0.32 mm [–0.23 to 0.87 mm]) or weight (–1.12 g [–7.31 to 5.07 g]). Causal mediation analysis showed the effect of ET on upper limb function was not mediated by proprioceptive ability.

**Conclusions:**

Upper limb function but not proprioception was impaired in ET. The effect of ET on motor function was not mediated by proprioception. These results indicate that the central nervous system of people with ET is able to accommodate mild to moderate tremor in active proprioceptive tasks that rely primarily on afferent signals from muscle spindles.

## Introduction

Essential tremor (ET) is the most common movement disorder and one of the most common neurological disorders [1,2], surpassing stroke, multiple sclerosis and epilepsy [3]. It is characterized by kinetic tremor in the hands and may be accompanied by other forms of tremor (*e.g*. postural, intention, resting) with other motor and non-motor features that reflect the cerebellar system degeneration characteristic of this disease [4]. Unfortunately, ∼75% of people diagnosed with ET experience activity limitations (*i.e*. disability) in tasks such as eating, drinking, writing and mobility [5,6,7]. While tremor amplitude is generally related to the severity of activity limitations experienced by people with ET (R^2^ from 0.28 to 0.7) [6,8,9,10,11], this is not always the case for postural tremor [11]. Moreover, tremor amplitude is not always related to self-reported tremor disability [11] or reduced quality of life [12]. This begs the question: do other deficits contribute to upper limb disability in ET?

Proprioceptive deficits are one possibility. Proprioception, which encompasses several senses, is required for normal human movement, and involves both central and peripheral structures and neural processes (for a review see Proske & Gandevia [13,14]). In the presence of tremor, afferent signals from proprioceptors located in muscles (e.g. muscle spindles), joints and skin will signal these involuntary oscillations. That is, tremor will add noise to afferent proprioceptive signals [15]. In the case of muscle spindles that have contractile elements, the presence of tremor-related oscillatory activity in fusimotor drive would directly impact their ability to signal muscle length and changes in muscle length. In line with this view, wrist position sense is impaired in people with dystonia and tremor, but not in people with dystonia alone [16]. Some aspects of proprioception, such as the senses of force and heaviness, involve central motor commands that may be contaminated by tremor-related rhythmic oscillations in people with ET [14,17,18,19,20]. Also, the central processing of proprioceptive signals involves various brain regions, including the cerebellum [21,22,23,24,25,26,27,28]. Given that ET is characterized by cerebellar system degeneration [4,20,29,30,31,32,33,34], proprioceptive deficits are likely to be present in people with ET and contribute to their upper limb disability. In support of this view, individuals with cerebellar damage have impaired proprioception when active muscle contractions are involved (e.g. the sense of force and heaviness), with the magnitude of proprioceptive deficits positively correlated with motor disability [35,36].

Despite the likely interplay between proprioception and tremor, as highlighted by Louis [37], few studies have investigated proprioceptive function in people with ET [38,39]. Moreover, new treatment options are needed for people with ET and other forms of tremor [40,41]. Thus, an important step forward is to better understand the sensory and motor deficits that contribute to functional limitations in these individuals.

To this end, the aim of the present study was to quantify the severity of proprioceptive deficits in ET and determine their contribution to functional impairments. We assessed upper limb sensory, proprioceptive and motor function, and administered a battery of upper limb functional tests in individuals with and without ET. Causal mediation analysis was used to estimate to what extent the effect of ET on a person’s upper limb function was mediated by their proprioceptive ability.

## Methods

### Participants

The two proprioceptive measures used in the present study have been shown to yield relatively precise estimates of between-group effects with sample sizes ranging from 10 to 21 [42,43,44]. Thus, we aimed to recruit ∼25 participants per group. Twenty-five participants with ET (values shown as mean (SD) unless otherwise stated; age: 57.1 (21.7) years, 12 females) and 24 healthy age- and sex-matched control participants (age: 60.0 (21.0) years, 15 females) were recruited. All participants were ≥18 years of age. Participants with ET met the diagnostic criteria in the Consensus Statement of the Classification of Tremors [45]. In addition, they had spectral plots of hand postural tremor and forearm electromyography (EMG) recorded under various loading conditions consistent with ET (electrophysiological outcomes 3 and 4 from Elble [46]). The medication regimen of participants was not altered for the experiment. Participants were excluded if they presented with muscle, joint or bone problems that affected their ability to perform daily tasks with the hands (*e.g*., rheumatoid arthritis, severe osteoarthritis, carpal tunnel syndrome), or if they had a neurological condition (*e.g*. Parkinson’s disease, stroke, multiple sclerosis).

Participants with ET were interviewed to determine how long they had been aware of their tremor, which parts of the body were affected, if there was a family history of tremor, and if they were taking medication for tremor. The more affected upper limb of participants with ET was tested, and the corresponding dominant or non-dominant upper limb was tested in the corresponding age- and sex-matched control participant. The study was approved by the University of New South Wales Human Research Ethics Committee (HC16918). All participants provided informed consent.

### Grip strength and tactile acuity

Maximal voluntary grip strength was measured in the test limb using a hand-held Jamar+ Digital Dynamometer (Lafayette Instrument Company, USA). Sitting in a straight-backed chair with feet flat on the floor and arms hanging down, participants maximally squeezed the handle of the dynamometer for 2–3 s as strong verbal encouragement was provided. Three trials were performed and the maximal value across trials was retained.

Tactile acuity was assessed with von Frey monofilaments [47] applied to the hypothenar eminence of the test limb. Monofilaments were slowly applied and removed over ∼2 s, and the skin was indented with a standardized force ranging from 0.008 to 300 g. The smallest force perceived was retained for analysis.

## Tremor assessment

### Clinical measures

Tremor was assessed clinically in all participants using Sections A (upper extremity section only, maximum severity = 24) and B (maximum severity = 36) of the Fahn-Tolosa-Marin Clinical Rating Scale (FTM) [48]. De-identified video images of participants performing scale items were assessed by a neurologist (DW). The Tremor Disability Questionnaire (TDQ) [49] was used with participants with ET to measure perceived functional limitation of daily activities due to tremor. We focused on the first 31 items of the questionnaire, which are used to tabulate a total hand tremor disability score, where higher scores indicate greater disability.

### Postural and kinetic tremor

Participants sat on a chair with the tested forearm supported on a table beside them. The shoulder was in ∼45° of abduction, the forearm was in pronation, and the wrist and hand were unsupported over the edge of the table. A firm strap secured the distal end of the forearm to the table. Thus, the set-up permitted full wrist flexion and extension while greatly limiting wrist pronation and supination.

Muscle activity was recorded with EMG using pairs of Ag-AgCl electrodes (Cleartrace; ConMed Corporation, Utica, NY, USA) placed over the muscle belly of the flexor carpi radialis (FCR) and the extensor carpi radialis (ECR) with an inter-electrode distance of 3 cm. A ground electrode was placed over the olecranon process.

**Figure 1:**
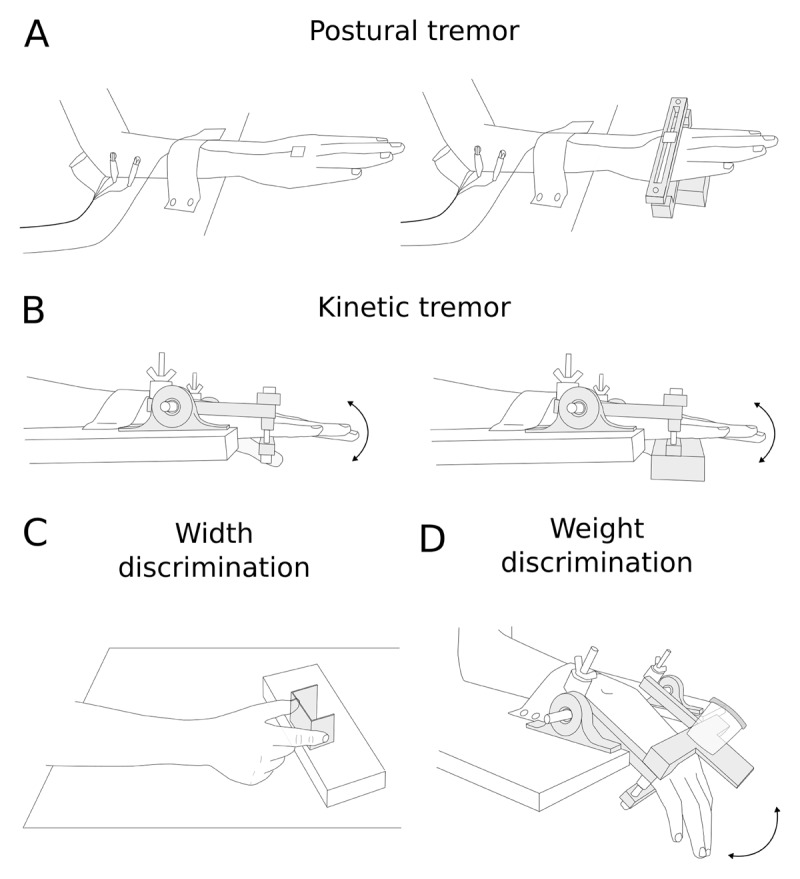
**Experimental set-up for tremor and proprioceptive testing.** **(A)** Experimental set-up to measure postural tremor without (left) and with (right) weight. An accelerometer was fixed to the dorsum of the hand, or on the device used to clamp the 300 g weight to the participant’s hand. Electromyography activity (EMG) was recorded with surface electrodes positioned over the extensor carpi radialis and flexor carpi ulnaris muscles (not shown). **(B)** Experimental set-up to measure kinetic tremor without (left) and with (right) weight. A magnetic rotary position sensor on the opposite side of the experimental set-up (not shown) captured the rotation of the experimental device. EMG was recorded from the extensor carpi radialis and flexor carpi ulnaris muscles (not shown). **(C)** Experimental set-up for width discrimination testing. **(D)** Experimental set-up for weight discrimination testing. The weights could be quickly and quietly slid onto and off from the dorsum of the device; they were held in place by a strong magnet embedded into the device.

Postural tremor was measured using an accelerometer placed on the dorsum of the hand, 1 cm proximal to the head of the middle metacarpal bone (MMA7361L, Freescale Semiconductor Inc.; ±1.5 g-force or ±6 g-force range, where g-force = 9.81 m/s^2^) (Figure 1A). Kinetic tremor was measured using a magnetic rotary position sensor (MLX90316, Melexis Inc.) with an experimental set-up similar to Héroux et al (Figure 1B) [50]. Briefly, the hand was clamped to a device at the palmar and dorsal aspects of the metacarpal heads. The clamps were connected to two shafts on either side of the wrist that rotated within low friction bearings, which allowed free flexion–extension movement of the wrist joint. A small magnetic disc fixed to the end of one of the shafts allowed the magnetic rotary position sensor to record angular displacement. All clamps, shafts, nuts and bolts were made of polylactic acid thermoplastic, and therefore the setup was light.

Postural and kinetic tremor were assessed with and without weights. For the weighted postural tremor condition, the hand was clamped on the palmar and dorsal aspect, which allowed a 300 g weight to be rigidly fixed. For the weighted kinetic tremor condition, the 300 g weight was fixed to the clamp that was part of the device used to measure kinetic tremor.

To assess postural tremor, participants held their wrist in neutral flexion-extension with fingers extended for 1 min with their eyes closed. To assess kinetic tremor, participants extended and flexed their wrist over 50° at a rate of 5°/s, starting with the wrist in 25° of flexion. Initially, participants looked at a monitor located 60 cm in front of them at eye height, where a target triangular waveform —two cycles of extension-flexion— was presented as well as a calibrated signal from the magnetic rotary position sensor.

Participants practiced slowly extending then flexing their wrist in synchrony with the triangular waveform; verbal feedback was provided to help participants maintain the target velocity. Once participants felt comfortable with the task, they closed their eyes and performed another two cycles of wrist extension-flexion, guided by verbal feedback from the experimenter. Visual feedback was not provided because, anecdotally, some individuals with ET report it worsens their tremor, and tracking a visual target introduces visuo-motor fluctuations in angular position in kinetic tremor testing [51]. One trial was recorded for each of the following conditions: postural unweighted, postural weighted, kinetic unweighted, kinetic weighted. Participants also performed two isometric maximal voluntary contractions (MVCs) with their wrist flexors and their wrist extensors. Each MVC lasted ∼2 s with a 60 s rest between contractions, and strong verbal encouragement was provided during the contractions.

Signals from the accelerometer and magnetic rotary position sensor were sampled at 200 Hz using Spike2 software with a 16-bit Cambridge Electronic Design (CED) 1401 plus data acquisition board. Muscle activity signals were amplified (gain x1000) and filtered (20 to 500 Hz bandpass, CED 1902, Cambridge, UK) prior to being sampled at 5000 Hz.

### Proprioception testing

We wanted to assess aspects of proprioception that might be impaired by tremor. Therefore, we focused on the ability to discriminate the width of objects actively grasped between the thumb and index finger (Figure 1C), and the ability to discriminate the weight of objects lifted with the wrist extensors (Figure 1D).

Discrimination thresholds –the smallest difference in weight or width that participants could detect in a given range– were determined using the transformed up-down staircase method with a two-alternative forced-choice procedure [52,53,54]. ‘Two-alternative forced-choice’ means that a reference stimulus and a test stimulus were presented in short succession in random order, and participants had to indicate which of the stimuli –first or second– was wider or heavier. ‘Transformed up-down staircase’ means that the width or weight of the test stimuli increased or decreased in fixed steps based on the number of correct or incorrect responses. Specially, we used a 1 up/2 down rule, where the test stimulus was made easier to discriminate (further in width or weight from the reference stimulus) after one incorrect response (*i.e*. 1 up) and harder to discriminate (closer in width or weight from the reference stimulus) after two correct responses (*i.e*. 2 down). We used a 2:1 ratio for the step sizes. That is, the step size to make the test stimulus easier to discriminate was two times bigger than the step size to make the test stimulus harder to discriminate. Details of the reference stimulus, the test stimuli and the step sizes used for width and weight discrimination are provided in their respective sections below.

For both width and weight discrimination, two staircases were simultaneously assessed: one staircase assessed widths or weights that were smaller than the reference stimulus, while the other staircase assessed widths or weights that were bigger than the reference stimulus. As testing progressed, trials were randomly selected from one of the two staircases. Each staircase ended after six reversals. A reversal is a trial where prior trials have steps in the same direction and subsequent trials have steps in the opposite direction. For example, if a participant had two correct responses in a row (correct, correct [step down]) followed by an incorrect response (incorrect [step up]), the last trial would constitute a reversal. Similarly, if a participant had an incorrect response (incorrect [step up]) followed by two correct responses (correct, correct [step down]), the last trial would constitute a reversal. Custom software written in Python (v3.4) was used to generate the staircases and record participant responses.

### Width discrimination

Participants were seated with the tested forearm and hand resting on a table beside them. The forearm was in neutral pronation-supination.

A 40 mm wide 3D printed rectangular object served as the reference stimulus, and a series of rectangular objects with widths ranging from 25 to 55 mm, in 1 mm increments, served as the test stimuli. One staircase started with participants comparing widths of 25 mm and 40 mm, while the other staircase started with participants comparing widths of 55 mm and 40 mm. The hand and all objects were concealed from view during testing. When participants made two correct judgments, the width of the test stimulus was incremented by 1 mm (*i.e*. step down, making judgment harder), whereas it was incremented by 2 mm when an error was made (*i.e*. step up, making judgment easier).

Each trial consisted of a series of three ‘open-pinch’ maneuvers: ‘open’ instructed participants to spread their index finger and thumb as wide as possible, and ‘pinch’ instructed participants to close their index finger and thumb to grasp the object being presented or to bring the index finger and thumb in contact with one another (Figure 1C). For the first ‘open-pinch’ maneuver, the reference object or the test object was presented; the order of presentation was randomized for each trial. For the second ‘open-pinch’ maneuver, participants pinched their index finger and thumb together. For the third ‘open-pinch’ maneuver, the object that remained (reference or test) was presented.

### Weight discrimination

Participants were seated with the tested forearm in pronation and supported on a table beside them. The hand was positioned in a device similar to that used to test kinetic tremor, except that this device had a rigid stop that limited wrist flexion to 30° and a top clamp on the dorsum of the hand that was fitted with a strong earth magnet. The rigid stop allowed participants to rest fully in between each active wrist extension, and the magnet allowed the reference and test weights to be secured to the dorsum of the hand (Figure 1D).

Weights were made using small plastic containers filled with metal ball bearings and foam. The foam was packed firmly to ensure the ball bearings did not shift during testing. In addition, metal washers were glued to the bottom of the plastic containers to ensure a strong hold with the magnet. The reference stimulus weighed 200 g and the test stimuli ranged in weight from 100 to 300 g, in 5 g increments. One staircase started with participants comparing weights of 100 g and 200 g, while the other staircase started with participants comparing weights of 300 g and 200 g. The hand, the testing device, and all weights were concealed from view during testing. When participants made two correct judgments, the weight of the test stimulus was incremented by 5 g (*i.e*. step down, making judgment harder), whereas it was incremented by 10 g when an error was made (*i.e*. step up, making judgment easier).

Each trial consisted of a series of two ‘lift’ maneuvers. For each lift, participants actively extended their wrist from 30° flexion to 30° extension, and lowered their wrist down. The reference object and the test object were randomly presented during either the first or the second ‘lift’.

### Upper limb function

The ABILHAND questionnaire [55] was used to measure perceived ability to perform daily activities that require the use of both hands. Participants with ET were instructed to score their manual ability based on when their tremor was most severe. The sum of raw scores was converted to log-odds probability units (i.e. logits), with lower logits indicating greater disability. The Box and Block Test [56] was used to measure unilateral gross manual dexterity. Participants were instructed to transfer wooden blocks (2.5 cm^3^), one by one, from one box compartment to another as quickly as possible for 1 min. The total number of blocks transferred was recorded, with higher values indicating greater gross manual dexterity. Finally, the 9 Hole Peg Test [57] was used to measure finger dexterity. Participants were instructed to transfer pegs from a container into holes on a board one by one as quickly as possible, then transfer them back to the container. Participants performed two trials following a short practice. The mean time (s) to complete the two trials was recorded, with lower times indicating greater dexterity.

### Data analysis

Acceleration and angular displacement signals were calibrated and band-pass filtered (0.5 to 40 Hz, 4^*th*^ order, dual-pass, Butterworth). Postural tremor during unweighted and weighted conditions was calculated as the root-mean-square of the acceleration signal. Kinetic tremor during unweighted and weighted conditions was calculated as the average root-mean-square of the angular displacement signal over the middle 40° of each of the 50° flexion and extension movements.

Muscle activity signals were mean-removed, filtered (20 to 450 Hz bandpass, 4*^th^* order, dual-pass Butterworth), rectified, and filtered again (40 Hz low pass, 4*^th^* order, dual-pass Butterworth). Maximal FCR and ECR EMG activity across the two MVC trials was determined and used to normalize the EMG recorded during postural and kinetic tremor testing.

Power spectra were computed for acceleration, angular displacement and EMG signals using the method of disjoint segments [58]. Spectra were estimated by averaging the finite Fourier transforms calculated over 12 non-overlapping windows of 5 s, resulting in a frequency resolution of 0.2 Hz.

To calculate proprioceptive discrimination thresholds, the absolute difference between the width or weight of the reference stimulus and the weight or width of each reversal (test stimuli) was first computed. Next, the initial reversal of each staircase was removed. Finally, the mean of the remaining 10 reversals (5 from each staircase) was computed to provide a discrimination threshold for width (in mm) and weight (in g).

### Statistical analysis

Robust regression was used to compare outcomes between groups using the *rreg* function in Stata (v13) in separate models, adjusting for grip strength and tactile acuity. Robust regression is more resistant to outlying observations (which are prevalent in participants with ET) than ordinary least squares regression methods, and allows 95% confidence intervals (CI) of between-group mean differences to be determined in natural units of the outcomes, without needing to log-transform the data [59]. If the 95% CI of the between-group mean difference crosses zero, we infer that there is no difference between groups.

Applying an approach from epidemiology, exploratory causal mediation analysis was conducted to estimate if the effect of ET on function was mediated by proprioception, with tremor amplitude as a second mediator. Causal mediation analysis aims to estimate effects that are mediated through specific pathways. Plausible mechanisms are specified in a directed acyclic causal graph, and adjustments are made to control for confounding [60,61]. The analysis then partitions the average total effect of an exposure on an outcome into the average effect acting through a mediator (the average causally mediated effect), and the average direct effect.

**Figure 2:**
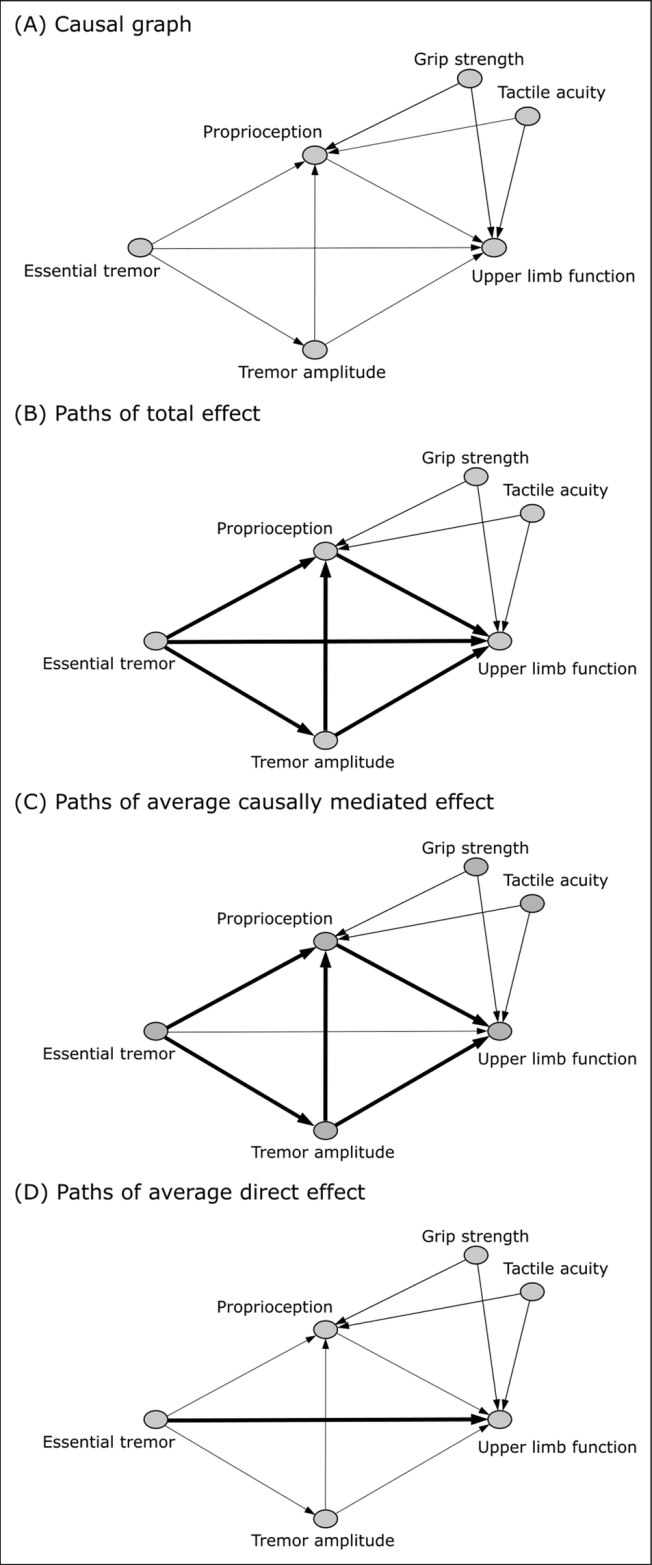
**Causal graph and highlighted paths of the plausible mechanism of essential tremor on function mediated by proprioception and tremor amplitude.** **(A)** Full causal graph with **(B)** paths of total effect highlighted, **(C)** paths of average causally mediated effect highlighted, and **(D)** paths of average direct effect highlighted.

A causal graph (Figure 2A) was developed by the investigator team using subject matter expertise, and generated using DAGitty software [62]. Arrows indicate the flow of causation between variables. Under the assumed causal graph, the effect of ET (exposure) on upper limb function (outcome) is mediated by proprioception and tremor amplitude, where the mediators are causally dependent. Grip strength and tactile acuity are potential confounders. Causal mediation analysis was performed using the *multimed* function within the mediation package in R (v3.6) [63,64]. This analysis partitioned the total effect (Figure 2B) into the average causally mediated effect (Figure 2C) and the average direct effect (Figure 2D), controlling for confounders. To reduce the number of comparisons and minimize Type I error, causal mediation analysis was conducted to estimate the effect of ET that is mediated by proprioceptive discrimination (width, weight), accounting for the effect of postural and kinetic tremor amplitude only measured under weighted conditions [50] on functional outcomes (*i.e*. Fahn-Tolosa-Marin Clinical Rating Scale for Tremor, ABILHAND, Box and Block Test, 9-Hole Peg Test) in separate models. For each outcome, we report estimates of the average causally mediated effect and the average direct effect (in participants with ET, and averaged between ET and control groups), and the total effect.

When mediators are causally dependent (Figure 2A), the average causally mediated effect assumes that interactions between the primary mediator (proprioception) and outcome (function) do not depend on the exposure (ET); this is known as the homogenous treatment assumption [63,64]. To assess the validity of this assumption, *multimed* was used to perform sensitivity analyses using bootstrapped simulation to generate separate sensitivity plots for participants with ET and control participants.

## Results

Twenty-four healthy control participants and 25 participants with ET took part in the study. Based on their medical history, EMG and tremor power spectra from weighted and unweighted postural tremor testing, and clinical tremor assessments (i.e. FTM), 7 participants were excluded (Controls: 2, ET: 5). The 2 control participants were outliers for postural tremor amplitude (>3 SD from group mean) and inspection of the power spectra revealed that they had spectral peaks characteristic of synchronized oscillatory muscle activity (wrist extensor EMG) and pathological tremor (acceleration). In 3 of the participants from the ET group, inspection of the power spectra revealed an EMG spectral peak that shifted to a lower frequency in the weighted condition, indicating that the tremor was likely not of central origin (*i.e*. it was possibly enhanced physiological tremor) [46]. The other 2 participants from the ET group had postural tremor amplitudes that, on the day of testing, were in the range of values for the control group. Inspection of their power spectra revealed no sharp peaks indicative of pathological tremor. One of these participants had a family history of ET.

In 15/20 participants with ET, the dominant hand was the more affected hand tested for proprioceptive abilities, and in 19/22 control participants the dominant hand was tested. In participants with ET, 35% (7/20) took medications for essential tremor. The mean (SD) time since the onset of noticeable hand tremor was 16.8 (13.0) years.

**Figure 3:**
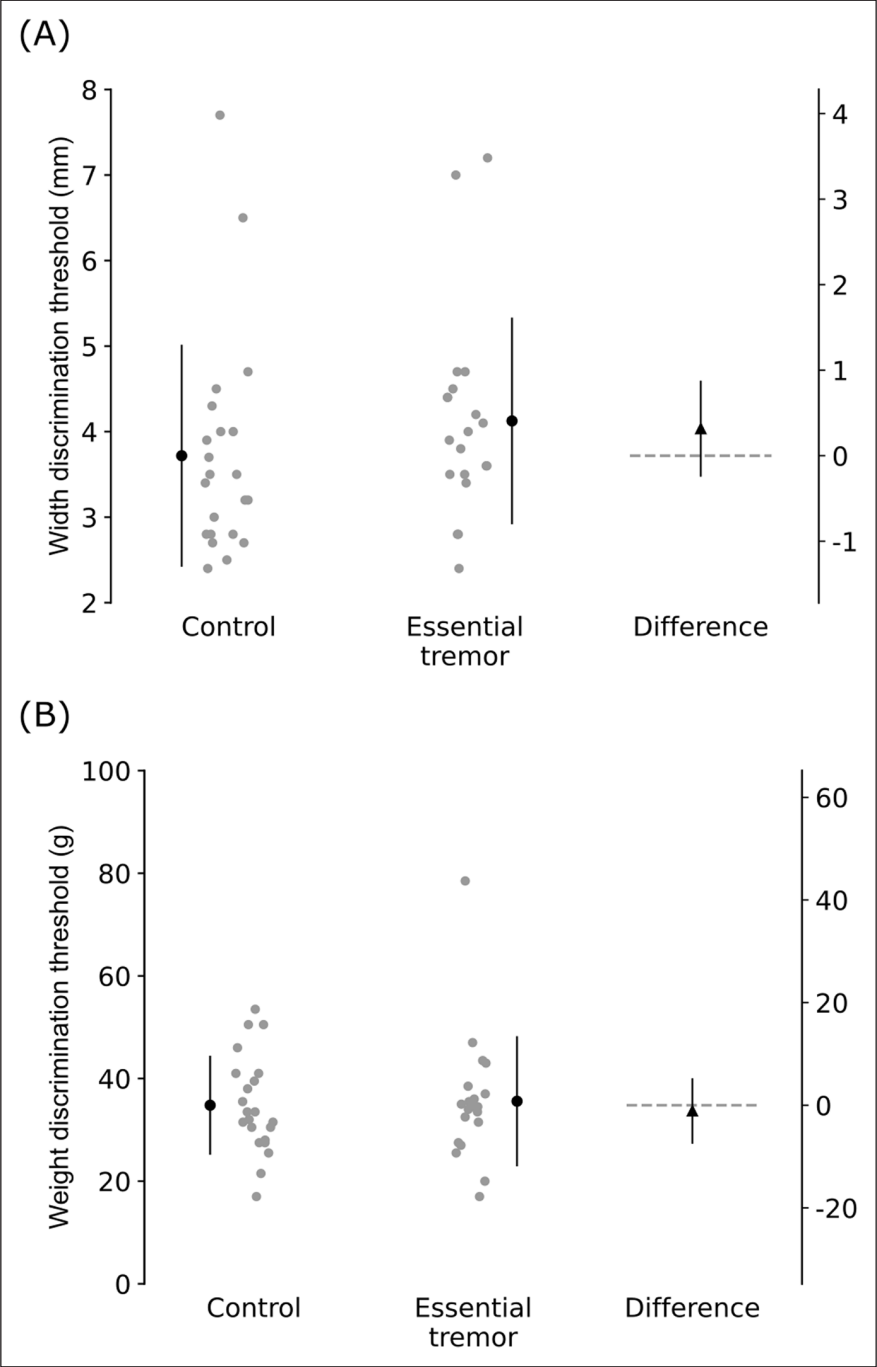
**Proprioceptive outcomes of (A) width discrimination threshold and (B) weight discrimination threshold.** Panels show individual participant data (gray circles), group mean and SD (black circle and error bar), between-group mean difference and 95% CI (black triangle and error bar), and the line of no between-group difference (dashed gray line).

Participants with ET had impaired upper limb function for all outcomes, and greater postural and kinetic tremor when compared to control participants (Table 1). However, there were no differences between groups in proprioceptive discrimination of width (mean difference [95%CI]: 0.32 mm [–0.23 to 0.87 mm]) or weight (–1.12 g [–7.31 to 5.07 g]; Figure 3).

**Table 1:** **Proprioception, tremor amplitude and functional outcomes.**


OUTCOME	CONTROL (n = 22)	ESSENTIAL TREMOR (n = 20)	BETWEEN-GROUP MEAN DIFFERENCE*	BETWEEN-GROUP MEAN DIFFERENCE (95% CI)†

Grip strength (kg)	32.4 (9.4)	34.8 (11.8)	2.3 (–4.3 to 8.9)	–

Tactile acuity (g)	0.17 (0.25)	0.32 (0.34)	0.14 (–0.05 to 0.33)	–

Postural tremor, unweighted (ms^–2^)	0.04 (0.01)	0.15 (0.14)	0.10	**0.03 (0.01 to 0.04)**

Postural tremor, weighted (ms^–2^)	0.05 (0.01)	0.20 (0.19)	0.16	**0.04 (0.03 to 0.05)**

Kinetic tremor, unweighted (deg)	0.36 (0.01)	0.65 (0.36)	0.30	**0.14 (0.06 to 0.23)**

Kinetic tremor, weighted (deg)	0.35 (0.10)	0.53 (0.20)	0.17	**0.11 (0.04 to 0.18)**

FTM-A (a.u.; max score 24)	1.0 (1.2)	5.6 (3.4)	4.2	**4.0 (2.6 to 5.4)**

FTM-B (a.u.; max score 36)	3.1 (2.8)	10.2 (5.0)	6.0	**6.3 (3.8 to 8.7)**

FTM-AB (a.u.; max score 60)	4.1 (4.0)	15.8 (7.8)	10.2	**10.5 (6.8 to 14.2)**

TDQ (%)	–	0.19 (0.11)	–	–

ABILHAND logits (a.u.)	5.11 (1.13)	2.90 (1.27)	–2.09	**–2.06 (–2.90 to –1.22)**

Box and Block Test (n)	69 (11)	58 (11)	–10	**–9 (–16 to –1)**

9-Hole Peg Test (s)	18.5 (2.9)	24.5 (6.5)	5.0	**3.9 (1.4 to 6.5)**


Within-group mean (SD) for all outcomes. Between-group mean differences (95% CI) are provided for grip strength and tactile acuity. For all other outcomes, between-group mean differences from linear regression (*) and between group mean differences (95% CI) for robust regression (†) are adjusted for grip strength and tactile acuity. Legend: a.u.: arbitrary units; FTM: Fahn-Tolosa-Marin Clinical Rating Scale; TDQ: Tremor Disability Questionnaire.

Causal mediation analysis showed the effect of ET on upper limb function was not mediated by proprioceptive discrimination of width or weight (Table 2). In general, sensitivity plots indicated that average causally mediated effects were not sensitive to the homogenous treatment assumption. That is, interactions between proprioception and function did not depend on whether participants had ET or not. Thus, under this causal graph, we can be confident that the effects of ET on function were not mediated by proprioception.

**Table 2:** **The effect of essential tremor on function that was causally mediated by proprioceptive discrimination (primary, 1°), where tremor amplitude was also a mediator (secondary, 2°).**


OUTCOME	1° MEDIATOR	2° MEDIATOR	ACME (ET)	ACME (AVERAGE)	ADE (ET)	ADE (AVERAGE)	TOTAL EFFECT

FTM (a.u.)	Width	Postural	–0.4 (–3.5 to 2.7)	–0.4 (–3.3 to 2.5)	10.6 (6.1 to 15.0)	10.6 (6.3 to 14.9)	10.2 (6.9 to 13.2)

	Width	Kinetic	0.1 (–1.2 to 1.3)	0 (–1.0 to 1.1)	10.2 (6.6 to 13.8)	10.2 (6.8 to 13.6)	10.2 (6.7 to 13.4)

	Weight	Postural	–0.4 (–3.2 to 2.4)	–0.4 (–2.9 to 2.0)	10.6 (6.5 to 14.8)	10.6 (6.5 to 14.8)	10.2 (6.9 to 13.3)

	Weight	Kinetic	–0.1 (–1.8 to 1.6)	–0.1 (–1.1 to 1.0)	10.3 (6.9 to 13.8)	10.3 (6.9 to 13.7)	10.2 (7.0 to 13.4)

ABILHAND logits (a.u.)	Width	Postural	0.03 (–0.71 to 0.76)	0 (–0.73 to 0.73)	–2.01 (–3.11 to –1.03)	–2.09 (–3.01 to –1.09)	–2.09 (–2.83 to –1.37)

	Width	Kinetic	0.05 (–0.23 to 0.32)	0.01 (–0.25 to 0.27)	–2.07 (–2.91 to –1.23)	–2.10 (–2.88 to –1.32)	–2.09 (–2.81 to –1.38)

	Weight	Postural	0.01 (–0.65 to 0.67)	0.01 (–0.63 to 0.65)	–2.10 (–3.03 to –1.18)	–2.10 (–3.02 to –1.19)	–2.09 (–2.83 to –1.36)

	Weight	Kinetic	0.02 (–0.32 to 0.36)	0.02 (–0.25 to 0.29)	–2.11 (–2.86 to –1.36)	–2.11 (–2.85 to –1.37)	–2.09 (–2.79 to –1.37)

Box and Block Test (n)	Width	Postural	–1 (–7 to 5)	–1 (–6 to 4)	–9 (–16 to –1)	–9 (–16 to –1)	–10 (–16 to –4)

	Width	Kinetic	0 (–3 to 3)	0 (–3 to 2)	–9 (–16 to –3)	–9 (–16 to –3)	–10 (–16 to –4)

	Weight	Postural	0 (–6 to 5)	0 (–6 to 5)	–9 (–17 to –2)	–9 (–17 to –2)	–10 (–16 to –4)

	Weight	Kinetic	0 (–2 to 2)	0 (–2 to 2)	–10 (–16 to –3)	–10 (–16 to –3)	–10 (–16 to –3)

9–Hole Peg Test (s)	Width	Postural	–0.1 (–2.4 to 2.1)	0 (–1.8 to 1.9)	4.8 (1.2 to 8.5)	5.0 (1.4 to 8.7)	5.0 (2.1 to 8.0)

	Width	Kinetic	–0.3 (–2.0 to 1.5)	0 (–1.1 to 1.1)	4.8 (1.4 to 8.2)	5.1 (1.8 to 8.4)	5.0 (2.2 to 8.0)

	Weight	Postural	0 (–3.3 to 3.2)	0 (–2.5 to 2.6)	5.0 (1.4 to 8.6)	5.0 (1.5 to 8.6)	5.0 (2.2 to 8.0)

	Weight	Kinetic	–0.1 (–2.6 to 2.3)	–0.1 (–1.4 to 1.2)	5.1 (2.1 to 8.1)	5.1 (2.2 to 8.1)	5.0 (2.2 to 8.1)


Only tremor amplitude during the weighted condition was analysed [50]. Estimates of the average causally mediated effect (ACME), the average direct effect (ADE) and all 95% CI were determined under the homogenous treatment assumption, adjusted for grip strength and tactile acuity. Mean total effects correspond to linear regression between-group mean differences (Table 1). For brevity, only the ACME and ADE for participants with ET and averaged between groups are shown. Legend: a.u.: arbitrary units; FTM: Fahn-Tolosa-Marin Clinical Rating Scale.

## Discussion

Essential tremor is a multifaceted neurological disorder associated with a variety of motor, sensory and cognitive deficits [10,65,66], the majority of which can contribute to activity limitations. Here we measured two distinct proprioceptive abilities, width and weight discrimination, in the more severely affected hand of people with ET and determined whether these proprioceptive abilities are causally linked to upper limb functional deficits.

In line with previous studies, our group of individuals with ET presented with kinetic tremor, postural tremor, and upper limb functional deficits [6,31,67,68]. While all participants with ET had tremor in the limb in which proprioception was tested, we found no evidence of proprioceptive deficits. That is, compared to the age- and sex-matched healthy participants, there was little to no difference on average in the ability of participants with ET to discriminate the width of grasped objects or the weight of lifted objects.

Why were proprioceptive deficits not present? Based on clinical disability and tremor scores [6,68], participants in the present study had mild to moderate ET. Since the severity of tremor and its relationship to functional limitations scales logarithmically in ET [6,67], it is possible that proprioceptive deficits in these tasks are only present in individuals with more severe ET. Although this may seem like the standard fallback argument to explain negative results in studies involving people with ET, it remains valid: there may be a limit to the amount of tremor-related proprioceptive noise on afferent signals from muscle spindles that the central nervous system can accommodate. This hypothesis should be confirmed (or refuted) by conducting a comparable experiment across the full range of tremor severity.

Another possibility is that the types of proprioceptive tasks tested in the present study (width and weight discrimination) are simply not impaired in ET. This would imply that tremor-related noise superimposed over both efferent and afferent neural signals does not impact the ability of people with ET to discriminate the width of a grasped object or the weight of a lifted object, tasks that rely on proprioceptive signals from muscles spindles [70,71]; receptors that should be affected by the rhythmic oscillatory motor drive characteristic of ET. In other words, the brain is able to compensate or account for the presence of this neural noise on afferent signals from muscle spindles when making these judgments.

To date, few studies have investigated the presence of proprioceptive deficits in people with ET. In their study, Semrau et al [39] found no proprioceptive deficits in individuals with severe tremor requiring deep brain stimulation. However, the joints they assessed –the elbow and shoulder– are less affected by tremor, and the experimental tasks required people to match the position or movement of a limb at rest, a state during which tremor is less likely to occur. Although not purely a test of proprioception, it is worth highlighting that people with ET and people with cerebellar degeneration both have difficulty distinguishing between two closely timed finger movements elicited by indwelling electrical muscle stimulation [38,69,72], a task that involves cerebellar neural computations [73,74].

A limitation of the present study is that we focused solely on traditional proprioceptive tests that involve relatively simple neural computations to detect, discriminate and match proprioceptive signals. There is a category of proprioceptive judgments that require proprioceptive-based central representations to be transformed into other frames of reference, which adds complexity and involves higher-level brain functions [21,22,23,24,25,26,27,28,75]. Future studies on proprioceptive deficits in ET could include both classes of proprioceptive tests.

To estimate how proprioceptive deficits contribute to functional limitations, causal mediation analysis provides an approach that is related to, but more informative than simple correlation analysis. This is because it estimates mediated effects under a plausible causal structure, adjusts for potential confounding, and tests the validity of assumptions using sensitivity analysis. We found that the effect of ET on upper limb function was not mediated by proprioceptive discrimination of width or weight. Overall, the 95% CI about the between-group mean differences for all outcomes were narrow, indicating we can be confident about our findings because estimates were precise.

Why did we perform causal mediation analysis if there was no between-group difference in proprioceptive abilities? The aim of this study was to determine the contribution of proprioceptive deficits to functional impairments in ET. If we adopted a traditional approach, we would have correlated proprioceptive ability with measures of upper limb function even though proprioceptive abilities were not different between groups. The absence of a meaningful difference in proprioceptive abilities between participants with ET and healthy controls would not have influenced whether correlations were performed. This is because the absence of a between-group difference does not indicate whether proprioceptive abilities are, or are not, related to upper limb function. Therefore, by extension, the absence of a between-group difference in proprioceptive abilities in the present study did not influence whether we carried out the planned causal analysis.

## Conclusion

People with ET presented with both kinetic and postural tremor, which was accompanied by impaired upper limb function. However, proprioceptive discrimination of width and weight were not impaired in these individuals, nor was the effect of ET on function causally mediated by proprioception.

## Abbreviations

average causally mediated effect, ACME; average direct effect, ADE; electromyography, EMG; essential tremor, ET; extensor carpi radialis, ECR; Fahn-Tolosa-Marin Clinical Rating Scale, FTM; flexor carpi radialis, FCR; maximal voluntary contraction, MVC; Tremor Disability Questionnaire, TDQ.
